# Retraction: MicroRNA-802 inhibits epithelial-mesenchymal transition through targeting Flotillin-2 in human prostate cancer

**DOI:** 10.1042/BSR-2016-0521_RET

**Published:** 2023-03-01

**Keywords:** epithelial-mesenchymal transition, Flot2, metastasis, microRNA-802, prostate cancer

This article is being retracted from *Bioscience Reports* at the request of the authors following receipt of a notification from a reader, alerting the Editorial Office to images that seem to appear in other papers by unrelated authors. The Figure 6 invasion/miR-Con panel seems to appear as the Figure 7F shRNA-NC panel from Yang et al. 2020 (https://doi.org/10.3389/fonc.2020.01104), and the Figure 5 GAPDH bands also seem to appear as the Figure 5F GAPDH bands from Liu et al. 2020 (https://doi.org/10.1042/BSR20191330). The authors were contacted regarding the concerns raised and provided the same cell image for the Figure 6 invasion/miR-Con panel as shown in the figure, as well as some western blot data; however, they were not able to provide any of the original raw scans for the western blots. Given that no additional area was visible in the provided cell image and that no original scans of the western blot data were provided, doubt still remains over the authenticity of the data. The authors therefore wish to retract the article, and the Editor-in-Chief and Editorial Board agree with the retraction.

